# Long COVID risk by pre-infection symptoms and functional status: A retrospective cohort study of data from the *All of Us* Research Program

**DOI:** 10.1101/2025.08.07.25333259

**Published:** 2025-08-12

**Authors:** Kristen Kehl-Floberg, Emma Freisberg, Aurora Pop-Vicas, Ron Gangnon, Dorothy Farrar Edwards

**Affiliations:** 1 Institute for Clinical and Translational Research, University of Wisconsin-Madison, Madison, Wisconsin, U.S.A.; 2 Department of Kinesiology, School of Education, University of Wisconsin-Madison, Madison, Wisconsin, U.S.A; 3 School of Medicine and Public Health, University of Wisconsin-Madison, Madison, Wisconsin, U.S.A.; #a Geriatric Research, Education, and Clinical Center (GRECC), William S. Middleton Veteran’s Memorial Hospital, 2500 Overlook Terrace, Madison, Wisconsin, 53705, U.S.A.; #a Department of Kinesiology, School of Education, University of Wisconsin-Madison, Madison, Wisconsin, U.S.A.; #c Currently unaffiliated.

## Abstract

**IMPORTANCE:**

Over seven million U.S. adults experience “long COVID”, or persistent health issues after COVID-19. Multiple guidelines recommend the inclusion of functional status in long COVID diagnostic criteria, but more evidence is needed to guide this recommendation. This study explores the adjusted odds of developing long COVID by pre-infection symptoms and functional status, and the feasibility of estimating functional status using health records data. DESIGN: Retrospective cohort study in a multicenter national longitudinal cohort of U.S. adults with history of COVID-19, using health records and survey responses through July 2022 (*All of Us* CDR 7.0).

**EXPOSURE(S):**

Pre-infection (−5 years) incidences of (a) at least one symptom common in long COVID, and (b) functional status, indicated by *All of* Us baseline survey responses and diagnostic/procedure/billing codes. Disease and demographic data covariates were included in the adjusted models.

**RESULTS:**

*N* = 65,464 met inclusion criteria (*n*=40,655 had post-infection occurrences of at least one symptom (long COVID group), while *n=*24,809 had none). Adjusted odds ratios within 99% confidence intervals [99% CI] of developing long COVID increased with lower pre-infection self-reported mental health (“Good” compared to “Excellent” AOR=1.14 [1.04,1.25], *P*>0.000), and more pre-infection symptoms (compared to the median of four, people with zero had much lower odds (AOR=0.15 [0.04,0.61], *P*=0.008). Adjusted odds were not significantly affected by any single pre-infection symptom, self-rated physical ability, or clinical documentation of functional impairment.

**CONCLUSIONS.:**

Greater pre-illness symptom burden and lower self-rated mental health increased the odds of long COVID symptoms, after adjusting for demographics, variant, functional status, and individual symptoms. Long COVID represents a change from baseline functioning and health, even in people with pre-infection incident symptoms and functional impairments. This estimation of pre-infection functional status using harmonized electronic health records data demonstrated the feasibility of these data in developing the diagnostic utility of functional status changes in long COVID.

## Introduction

Among U.S. adults with a history of SARS-CoV-2 infection, 7.7 to 23 million experience persistent post-acute symptoms [1] termed “long COVID” [2]. About 7.5% of U.S. adults reported long COVID symptoms in June 2024, and about 5.5% reported activity limitations as a result [3]. Long COVID is a patient-generated term for new-onset or exacerbated, and persistent, symptoms and health conditions after COVID-19 infection [2, 4–8]. Common presentations include fatigue, post-exertional malaise, cognitive impairment, headache, breathing difficulty, musculoskeletal pain or weakness, sleep disturbance, and mood disorder [9] among many others [10–12]. Long COVID symptoms affect complex and “chosen” activity patterns (such as productivity and leisure) more dramatically than basic self-care [13, 14] forcing some patients to abandon complex routines and roles in work, family, and community life [6, 15].

Clinical diagnostic definitions and criteria of long COVID are an urgent and ongoing focus of study [2]. Multiple research initiatives have been launched to study recovery from COVID-19 in large samples with the goal of identifying unique signatures of symptoms and biomarkers [10, 16–21], many using electronic health records (EHR) data. These studies have marshalled extraordinary amounts of data into various phenotypes and diagnostic hallmarks [22], providing vital direction on the disease and its impact. However, few have included quantifiable indicators of daily functioning [23], despite early patient reports describing profound impact on daily life [6, 24] and proposed criteria asserting that an impact on daily functioning is a critical diagnostic component [2, 25]. Because most EHR-based phenotyping studies have not included data on how disability manifests or is treated, their resulting diagnostic classifications have excluded this diagnostic feature.

Additionally, although post-infection presentations have been thoroughly investigated, studies of pre-infection occurrences of these same symptoms, conditions, and functional impairments are limited [16, 26]. Most studies that explore the emergence of symptoms and conditions after COVID-19 have not compared this to, nor assessed risk based on, their pre-infection prevalence [22]. Concerning functional status, pre-infection disability has been found to be associated with a two- [27] to three-fold [28] increase in long COVID. Because long COVID includes worsened or exacerbated conditions that were previously well-managed [5], pre-infection symptoms and functional status may be relevant to both estimating risk of developing long COVID and tailoring management strategies [22].

The primary aim of this study is to compare pre-infection functional status, pre-infection occurrences of common long COVID symptoms, and demographics, between participants with and without long COVID in a diverse population-based sample. Our secondary aim is to estimate the adjusted odds of developing at least one long COVID condition based on these pre-infection characteristics.

## Materials and Methods

### Design and data source

This is a retrospective cohort study of participants enrolled in the U.S. National Institutes of Health *All of Us* Research Program through July 2022 (curated data repository version 7.0), Controlled tier C2022Q4R9 [29] accessed through the *All of Us* Researcher Workbench [30] between May 1 2023 and June 31 2025. *All of Us* is among the most comprehensive population-level biobanks in the world [31] enrolling participants from all 50 U.S. States and three territories and over-recruiting from groups that are historically underrepresented in medical research. They share baseline function and health data through surveys, biospecimens, and release of EHR data which is subsequently scraped every three months. The program and data snapshots can be viewed at https://www.researchallofus.org/data-tools/.

### Sample

*All of Us* participants 18 years of age and older with at least one incidence of any indicator of COVID-19 illness were included. Diagnostic indicators were (a) laboratory values for positive PCR, antigen, or antibody test, (b) self-reported illness reported on the 2021–2022 *All of Us* COVID-19 Participant Experiences (COPE) survey, or (c) the ICD-10 code for COVID-19 (U07.7) (S1 Appendix A, Table A.1). Informed consent for sharing EHR and survey data was obtained from all participants by *All of Us* Health Provider Organizations responsible for participant recruitment and enrollment. IRB oversight was delegated to the *All of Us* Central IRB. Controlled tier data includes unique participant identifiers and dates; investigators transformed these (e.g. date of birth to years of age) promptly following import, and collapsed counts of fewer than 20 to prohibit subgroup membership identification in summary tables. These steps are in compliance with *All of Us* data use agreement policies.

### *Long COVID* and *Recovered* participant groups

Participants with COVID-19 history were grouped by whether their EHR showed at least one diagnosis or observation code from a list of 38 long COVID symptom and condition categories (see below, “Pre- and post-infection incidences of long COVID symptoms”, and S1 Appendix A, Table A.2) at least 28 days after first infection. Participants with at least one symptom were classified in the “long COVID” group; the rest were classified in the “recovered” group. (S1 Appendix A).

### Data sources and variables

#### Surveys: Demographics, self-reported health, and self-reported function at study enrollment.

*All of Us* participants provide self-report of their health and daily functional status data through validated self-report measures from the PROMIS battery and *All of Us*-designed surveys at enrollment. This study used responses to “The Basics” (demographics), “Overall Health” (daily functioning and mental health) and “Covid-19 Participant Experience” (COPE) (self-reported COVID-19 symptoms between May 2020 – March 2021) surveys.

#### Pre- and post-infection incidences of long COVID symptoms and conditions.

Symptoms of long COVID were chosen a-priori based on the Centers for Disease Control and Prevention’s list of symptoms [32] and emergency billing code for Post-COVID conditions [33], prior phenotyping and patient-led literature, and the authors’ clinical and community research experience. These sources yielded a list of 44 symptoms/conditions (“descendent” codes), which fell under 38 categories (“ancestor” codes) within the *All of Us* data table structure (S1 Appendix A, Table A.2). These 38 ancestor diagnoses were queried for all participants. For each category, the first incidence in the five years prior to infection signified a pre-infection occurrence, and the first incidence 28 days or more after the infection signified a post-infection occurrence (long COVID group only) of any symptom under that category. Because proposed empirical definitions of long COVID include exacerbations or relapses of pre-infection conditions [5, 11, 22], we queried “any mention” of the symptoms/conditions in this list (rather than “first mention”). Symptom persistence was not estimated due to the *All of Us* data structure and privacy policies. S1 Appendix A reports selection process, rationale, condition categories, and exclusions.

#### Total number of long COVID symptoms/conditions present prior to infection

From the pre-infection symptom occurrences, a gross measure of pre-infection symptom burden was computed for each participant by tallying all long COVID symptom categories with observations. This included all symptoms (e.g. headache, joint pain, cough) and conditions (ME/CFS, POTS, and post-viral fatigue syndromes) used in cohort discovery.

#### Infection variants active at time of first infection

A time-bound variable was created to infer variant exposure at date of first SARS-CoV-2 infection. Periods began and ended around the emergence of major variants as reported by Markov et al [34]. Our variant periods were “pre-VOC/wild type” from January-November 2020, “pre-VOC/alpha/beta from November 2020 – April 2021, “alpha/beta/delta” from April-August 2021, “delta” from August-December 2021, “omicron_BA1-BA2” from December 2021 – April 2022, and “omicron_BA2-BA5” from April 2022 – July 2022 (data release cut-off date).

#### Demographics

Age, sex assigned at birth, race, and ethnicity were included based on evidence that these factors influence risks for long COVID [35–38] and for functional impairment [39, 40]. Level of education was included as a proxy for social determinants of health due to its impact on access to health-supporting societal resources [41], as financial concerns [42] and greater unmet social needs [43] have been found to be associated with more numerous and persistent long COVID symptoms (S1 Appendix D, Table D.1).

#### Pre-COVID daily functioning

Pre-infection functional status was explored using enrollment surveys and functional status findings (EHR procedure and diagnostic codes) recorded between January 1, 2015 and four weeks prior to first infection. Survey responses were queried for self-reported performance of physically demanding daily activities, social roles, and mental/cognitive health. From the EHR, the incidences of medical billing codes for therapeutic procedures provided by occupational therapists were tallied [44] (S1 Appendix B). Additionally, procedure codes under the vocabulary hierarchy “Finding of Functional Performance and Activity” were grouped into three levels of presumable impairment (“None”, “Some”, and “Dependent on others”). (S1 Appendix B.)

#### Quantitative bias analysis of cohort discovery

Potential cohort bias was examined using non-parametric propensity score matching. Bias was estimated for participant enrollment before first COVID infection (retained for this analysis) versus after (excluded) and a 90 day symptom start date instead of 28 days (S1 Appendix C).

#### Analysis

Demographics of the long COVID and recovered groups were graphed and tested for significant difference using Welch’s *t* test (normal), Mann-Whitney U test (non-normal), or *X*^2^ test (categorical). Unadjusted odds ratios were computed for all variables. Logistic regression was used to model the adjusted odds of developing long COVID based on demographics, variant, pre-infection symptoms/conditions, and pre-infection functional status (S1 Appendix D, Table D.1-D.2, Figure D.1). Data access and analysis were performed in the *All of Us* Researcher Workbench, Jupyter notebooks cloud computing environment, using R [45]. Cohort discovery, data cleaning and organization, and statistical analyses were completed by the first author using R packages “stats” [46], “rms” [47], “corrplot” [48], and the “tidyverse” suite [49].

## Results

**Fig. 1.**
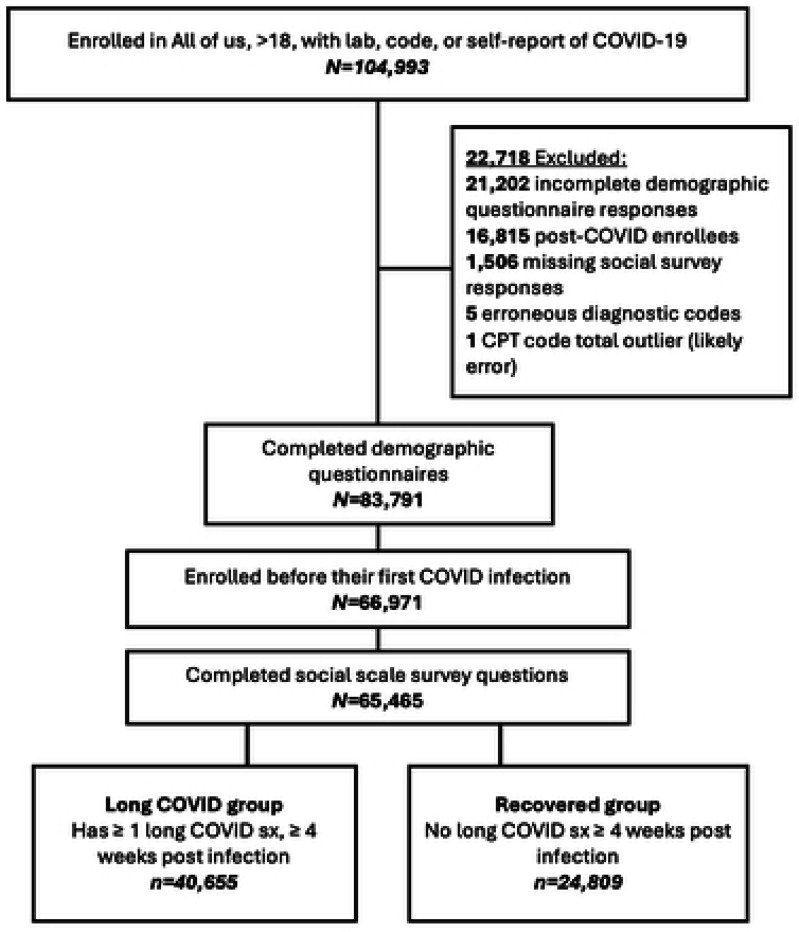
CONSORT diagram of sample flow.

104,992 participants met inclusion criteria of at least one COVID-19 illness. Participants with missing demographic survey responses (*n*=21,202) were removed. Further removed were by *n*=16,815 participants who enrolled after their first COVID-19 indicator (allowing use of enrollment questionnaires as pre-infection self-reported baseline functioning and health data); *n*=1,506 due to non-responses to the social survey questions (permitting these responses to be combined as an ordinal summary scale); *n=*5 participants with erroneous diagnostic codes (concept name and code was for SARS-CoV-2, but the dates of diagnosis corresponded with previous SARS-type virus outbreaks in the early 2000’s); and *n*=1 due to an extreme and clinically unlikely value of 1286 (or 321 hours) for their 5-year CPT billing code total. The final sample was *N* = 65,464 participants, with 40,655 showing at least one post-infection symptom (long COVID group) and 24,809 showing none (recovered group) (Figure 1).

### Quantitative bias analysis of cohort discovery

Non-parametric propensity score matching showed a low match rate of 45% between pre- and post-infection enrollees (S1 Appendix C, FigureS C.1-C.2). Changing the classification timeline to 90 days after first infection had a very small impact on the numbers of long COVID versus recovered participants (S1 Appendix C, Figures (C.3-C.4).

### Demographics

Demographic distributions, univariate unadjusted odds ratios and multivariate adjusted odds ratios are shown in Table 1. Most participants had their first infection during the pre-variant/wild type period, during which the proportion of long COVID participants outnumbered that of recovered participants by almost 2 to 1 (S1 Appendix E, Figure E.1). The Long COVID participants were older (median 63 (24) versus 60 (28)), and had slightly higher percentages of female and Black participants than the recovered group. Adjusted odds of developing long COVID after infection increased with older age, female sex, Black racial identity, and an earlier variant period of first infection. Groups were effectively equivalent in numbers of participants reporting Hispanic/Latino ethnicity.

### Pre-infection daily functioning

EHR-recorded billing code indicators of daily functioning showed higher proportion of performance difficulty in the long COVID group (10% vs. 4%) and dependence on others for care (6% vs. 2%). Participants in the long COVID group also more frequently selected lower ratings on baseline physical activity ability, social score, and mental health. However, when adjusted for demographics and pre-infection symptom occurrences, only lower self-rated mental/cognitive health increased the odds of developing long COVID (Table 1).

### Pre-infection occurrences of common long COVID symptoms and conditions

**Fig. 2.**
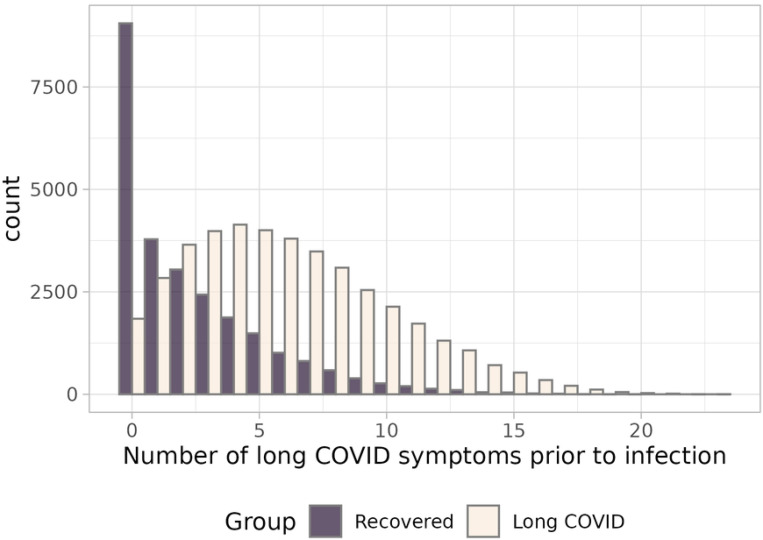
Number of long COVID symptoms with at least one pre-infection occurrence, by group. A histogram with side-by-side light (long COVID group) and dark (recovered group) bars. Shows the differences in distributions of the total number of long COVID symptoms experienced in each group *before* infection (between 5 years and 28 days). The *Y* axis plots the number of participants from zero to over 7,500; the *X* axis plots the range of number of pre-infection symptoms observed in this cohort, from zero to 23. The long COVID group shows a right-skewed normal curve (mean=6.11 (3.96)), while the recovered group shows an exponential distribution (mean=2.44 (2.93)).

The range of pre-infection occurrences of common long COVID symptoms was between 0 and 23. The long COVID group had a higher median number of pre-infection (6(6) vs. 1(4), Table 1, Figure 2). The proportions of the number of participants with any observations under these broad categories prior to infection are shown in Table 2 and S1 Appendix E, Figure E.2. There were fewer than 500 incidences of anosmia with sparse cells by group, so this symptom was not included in the model. There were no pre-infection observations of POTS, PVFS, ageusia.

Correlations were computed to test the pre-infection symptom categories for collinearity (S1 Appendix E, Table E.1) and grouped by first principal components analysis (Figure E.3). Depression and anxiety had the highest correlation (*r*=0.53). Moderate to low correlations were found between abdominal pain and both diarrhea (*r*=0.32) and chest pain (*r*= 0.32), dyspnea with both chest pain (*r*= 0.4) and cough (*r*=0.33), ME/CFS and fatigue (*r*=0.34), musculoskeletal and generic chest pain (*r*=0.33), and sleep and depression (0.31).

Having zero pre-infection occurrences of any common long COVID symptom/condition was associated with significantly lower adjusted odds of long COVID (AOR: 0.15, 99% CI: 0.04, 0.61, *p*=0.005). No single symptom/condition category increased the adjusted odds of developing long COVID. Sleep disturbance was the only symptom category that approached significance (*p*=0.02).

## Discussion

This retrospective cohort study is among the first examinations of long COVID among participants in the *All of Us* Research Program. To our knowledge, it is also the first population-level study to explore the prevalence of pre-infection occurrences of long COVID symptoms and functional status levels, and their associations with developing post-COVID symptoms.

These associative findings contribute several insights into the evolving understanding of the risks of long COVID. First, adjusting for demographics, pre-infection functional status, and pre-infection occurrences of common long COVID symptoms resulted in an overall shift in effect size to smaller or null differences between the long COVID and recovered groups. Nearly all variables were associated with significant unadjusted odds, suggesting that people with long COVID symptoms after infection were more likely to be older, female, Black or African American, have less education, more symptoms, lower pre-infection functional status, and lower self-rated health and function. However, in the full model and holding all covariates at their most-frequently observed values, only older age, Black or African American racial identity, female biological sex, variant period at first infection, and lower self-rated mental health were associated with increased odds of long COVID. These demographic findings are consistent with other large studies that have found higher prevalence of long COVID by race [35, 37], older age [36], and female biological sex [35, 36, 38].

This was the first study exploring the role of pre-infection functional status in a large dataset. In unadjusted odds, we noted different strengths and directions of associations from the self-reported versus EHR-derived functional status indicators (with lower pre-infection self-rated mental health/cognition being associated with developing long COVID, while ability to perform physical daily activities was not). This was somewhat surprising since many of the concepts contained in the “Finding of Functional Status and Activity” code hierarchy describe physical (e.g. motor and mobility) performance, with comparatively few cognitive or behavioral observations. This discrepancy could be due to different latent constructs being measured, or under-detection of slight or moderate functional decreases in EHR data [50]. In the adjusted odds, the only self-rating that retained a significant association with long COVID was a middle (“Good”) rating of cognition and/or mood. In contrast, the EHR-derived diagnostic codes for cognitive and mood disorders were non-significant. Self-reported functional impairments in long COVID are sometimes dismissed [6] or measured with instruments that haven’t been validated with this population [51, 52]. Exploring change from pre- to post-infection using instruments with relevance to the patient experience may provide more accurate insights.

This exploration of the pre-infection incidences of symptoms and conditions common in long COVID showed several insights. First, the distributions of these conditions relative to one another were remarkably similar between long COVID and recovered participants; with a few exceptions of slight magnitude, the conditions that were more prevalent in participants who later developed long COVID were also more prevalent in participants who later recovered. However, there was a striking difference in the *proportion* of each group experiencing each symptom, often twice as high or more in those who later developed long COVID. Second, no single symptom contributed significantly to higher adjusted odds of long COVID; only sleep disorders approached significance. From a diagnostic perspective, this aligns with patient-led research findings that long COVID is a change in health status from pre-infection including both new and exacerbated symptoms and conditions compared to pre-COVID, and that symptom presentation can vary widely between individuals [5, 12, 22]. Many (but not all) participants who developed long COVID experienced pre-infection health issues found in long COVID phenotypes, with a wide range of pre-infection symptom burden. Although the risk of long COVID was lower with no pre-infection symptoms, it was not eliminated. Additionally, although pre-infection symptom burden increased the unadjusted odds of long COVID, this association was not significant (except at zero symptoms) when adjusted for demographics, individual symptoms, and pre-infection daily functioning. Thus, prior symptom burden was neither necessary nor sufficient to support a singular causal link. Nevertheless, the assessment of pre-infection health, including the presence of and interaction between ongoing health needs, should be considered in research and clinical management of long COVID, as previously effective management strategies may require adjustment. A future analysis of long COVID participants could explore pre- versus post-infection changes in symptom and condition distribution, exacerbations versus new conditions, and dimensional reduction to explore and compare phenotypes.

The finding that earlier periods of first infection increased the likelihood of developing long COVID (S1 Appendix E) calls for additional context and exploration. Presumably, the opportunities to develop symptoms should have increased with time. This dataset’s cut-off date of July 2022 left little time for people in the last variant period to develop symptoms, and we did not control for time from first infection to first symptom. Additionally, there is evidence that vaccination against COVID-19 has some protective effect against long COVID [16, 53]. Thus, both the date at first infection and the availability of vaccines starting in 2021 may have influenced the number of people who developed symptoms. This may be explored in a future analysis.

### Limitations

This analysis contains several limitations. First, retrospective studies using EHR data are limited by detection bias [50, 54]. Several sources of relevant functional and disease severity data are unavailable in *All of Us*, and we were unsuccessful in finding proxies for severity of infection or long COVID symptoms. In studies of COVID-19 there is difficulty verifying that participants without COVID-19 diagnostic and lab indicators were truly unexposed, particularly when using retrospective EHR data. It is possible that many participants who were not selected for this sample based on EHR evidence of COVID-19 infection had, in reality, been ill with the virus but were not identifiable as such because they did not report their illness or present for care. Additionally, admitting diagnoses and narrative notes (where functional status is often described) are suppressed in *All of Us* for privacy, which precluded connections between incidences of COVID-19 and hospitalizations and the use of text-to-data methods of data extraction. Use of Remdesivir, used in acute treatment, was explored as proxy for disease severity but was not used. The cut-off date for this *All of Us* data set predated FDA approval of Remdesivir by six months. About 800 participants had been prescribed remdesivir, presumably for experimental or off-label treatment. This relatively small number was insufficient to make inferences across the sample, and may have been confounded by access to clinical trials. Prospective studies using tools that measure disease and symptom severity, as well as the patient experience, continue to be critical.

## Conclusions

Long COVID was more likely for participants with self-reported lower mental or cognitive health prior to infection. It was less likely for participants with no common long COVID symptoms occurring before infection, however no single long COVID symptom prior to first infection affected the odds of long COVID after adjusting for demographics, variant, and functional status. Long COVID represents a change from baseline functioning and health, even in people with pre-infection incident symptoms and functional impairments. Functional status indicators exist in harmonized electronic health records data and have the potential to reveal the functional impacts of long COVID thus improving diagnostic efficiency. Areas of future study include developing the diagnostic utility of functional status assessment, uncovering the relationship between long COVID-related functional changes and ageing, and the changes from pre-infection to post-infection for people with post-COVID symptoms.

## Supplementary Material

Supplement 1

## Figures and Tables

**Table 1.** Demographic, functioning, and long COVID disease characteristics and odds ratios of developing long COVID in *All of Us* participants through July 2022.

	Long COVID Symptoms	Recovered	*p*	OR	AOR
**n (total 65,464)**	40,655	24,809			
**age (mean (SD))**	61.01 (16.03)	57.66 (16.82)	< 2.2e-16		
25				0.54 (0.43, 0.69)	0.68 (0.59, 0.77), *p*>0.000
35				0.64 (0.59, 0.70)	0.75 (0.68, 0.83), *p*>0.000
45				0.73 (0.68, 0.79)	0.83 (0.79, 0.89), *p*>0.000
55				0.94 (0.87, 1.02) *p*<0.059	0.93 (0.91, 0.95), *p*>0.000
62				1.00	1.00
75				1.09 (1.02, 1.17)	1.15 (1.10, 1.20), *p*>0.000
85				1.21 (1.12, 1.31)	1.28 (1.18, 1.39), *p*>0.000
**Total number of pre-pandemic symptoms of long COVID (mean (SD))**	6.11 (3.96)	2.44 (2.93)	< 2.2e-16		
0				0.13 (0.12, 0.14)	0.15 (0.04, 0.61) *p*=0.008
1				0.47 (0.44, 0.51)	0.38 (0.13, 1.11) *p*=0.076
4				1.00	1.00
7				2.12 (1.99, 2.27)	1.61 (0.55, 4.68) *p*=0.381
23				4.78 (4.44, 5.16)	1.99 (0.00, 1703.58) *p*=0.842
**Variant of Concern**			< 2.2e-16		
Pre-VOC/wild type	21120 (51.9	7415 (29.9)		1.00	1.00
Pre-VOC, alpha, and beta	9890 (24.3)	6306 (25.4)		0.55 (0.52, 0.58)	0.61 (0.57, 0.65), *p*>0.000
alpha, beta, and delta	3240 (8.0)	1890 (7.6)		0.60 (0.56, 0.65)	0.48 (0.44, 0.53), *p*>0.000
delta	3462 (8.5)	2878 (11.6)		0.42 (0.39, 0.46)	0.33 (0.30, 0.36), *p*>0.000
omicron BA1-BA2	2568 (6.3)	3543 (14.3)		0.25 (0.24, 0.27)	0.17 (0.16, 0.19), *p*>0.000
omicron BA2-BA5	375 (0.9)	2777 (11.2)		0.05 (0.04, 0.06)	0.02 (0.02, 0.03), *p*>0.000
**Sex assigned at Birth (%)**			< 2.2e-16		
Female or intersex	26646 (65.5)	15287 (61.6)		1.18 (1.13, 1.24)	1.06 (1.01, 1.13), *p=*0.005
					
**Race (%)**			< 2.2e-16		
Asian	973 (2.4)	984 (4.0)		0.62 (0.55, 0.69),	0.92 (0.79, 1.10), *p*=0.14
Black or African American	8941 (22.0)	4645 (18.7)		1.20 (1.14, 1.26),	1.12 (1.05, 4.18),, *p*>0.00
Middle Eastern or North African	285 (0.7)	199 (0.8)		0.89 (0.70, 1.136), 0.21	1.03 (0.76, 1.40), *p*=0.80
More than one population	783 (1.9)	526 (2.1)		0.93 (0.80, 1.07), 0.18	1.01 (0.84, 1.20), *p*=0.88
White	39716 (97.7)	18455 (74.4)		1.00	1.00
**Ethnicity (%)**			0.00018		
Hispanic or Latino	939 (2.3)	689 (2.8)		0.83 (0.73, 0.94)	0.88 (0.75, 1.03), *p*=0.041
**Education (highest grade completed)**			< 2.2e-16		
Advanced Degree	9722 (23.9)	7230 (29.1)		0.7 (0.66, 0.73)	0.97 (0.90, 1.05), *p*=0.343
College Graduate	9654 (23.7)	6557 (26.4)		0.76 (0.72, 0.80)	1.02 (0.94, 1.09) *p*=0.585
Some College	11771 (29.0)	6104 (24.6)		1.00	1.00
High School/GED	7043 (17.3)	3658 (14.7)		0.99 (0.94, 1.06)	0.96 (0.89, 1.05) *p*=0.261
Some High School	1592 (3.9)	851 (3.4)		0.96 (0.86, 1.06)	0.85 (0.73, 0.99) *p*=0.005
Middle School	286 (0.7)	122 (0.5)		1.16 (0.91, 1.48)	1.02 (0.72, 1.46) *p*=0.866
Elementary	<40 (<0.1)	<40 (<0.1)		1.09 (0.55, 2.17)	0.72 (0.29, 1.77) *p*=0.342
No Formal Education	<40 (<0.1)	<40 (<0.1)		1.03 (0.25, 4.30)	0.84 (0.13, 5.26) *p*=0.801
No Answer	545 (1.3)	266 (1.1)		1.11 (0.93, 1.31)	1.04 (0.81, 1.33) *p*=0.683
**Pre-infection functional performance categories** **			< 2.2e-16		
No Functional Performance Difficulty	33933 (83.5)	23494 (94.7)		1.00	1.00
Some Functional Performance Difficulty	4106 (10.1)	926 (3.7)		3.07 (2.79, 3.38)	1.01 (0.62, 1.63) *p*=0.976
Dependent on Others for Care	2616 (6.4)	389 (1.6)		4.65 (4.05, 5.37)	1.38 (0.84, 2.27) *p*=0.096
**Pre-infection OT CPT** **codes billed (median IQR))**	0 (1)	0 (0)	*p* < 2.2e-16		
**mean (SD))**	4.45 (17.78)	2.06 (10.22)			
0				1.00	1.00
5				1.85 (1.71, 2.00)	0.99 (0.99, 1.01), 0.479
20				2.14 (1.95, 2.34)	0.99 (0.95, 1.03), 0.479
80				2.29 (2.04, 2.57	0.96 (0.82, 1.12), 0.479
700				3.62 (2.35, 4.65)	0.68 (0.17, 2.76) 0.479
**Self-rated ability to complete physical daily activities**			< 0.000		
Completely	21740 (52.2)	16608 (66.9)		1	1
Mostly	7715 (19.0)	3821 (15.4)		1.56 (1.47, 1.65)	1.05 (0.97, 1.13) *p*=0.128
Moderately	6415 (15.8)	2542 (10.2)		1.95 (1.83, 2.09)	1.02 (0.93, 1.12) *p*=0.547
A little	4123 (10.1)	1451 (5.8)		2.20 (2.02, 2.39)	0.95 (0.84, 1.07), 0.228
Not at all	661 (1.6)	254 (1.0)		2.01 (1.66, 2.44)	0.83 (0.64, 1.06), 0.046
No Answer	271 (0.7)	133 (0.5)		1.53 (1.20, 2.08)	1.00 (0.72, 1.42), 0.949
**Self-rated social role performance and satisfaction (composite measure)**			< 2.2e-16		
2	5197 (12.8)	3955 (15.9)		0.91 (0.85, 0.97)	1.04 (094, 1.10) *p*=0.326
3	5259 (12.9)	3921 (15.8)		0.93 (0.86, 0.99) *p*=0.004	1.03 (0.94, 1.10) *p*=0.433
4	8881 (21.8)	6129 (24.7)		1	1
5	6424 (15.8)	3728 (15.02)		1.19 (1.11, 1.37)	1.06 (0.98, 1.20) *p*=0.066
6	6668 (16.4)	3481 (14.03)		1.32, (1.23, 1.42)	1.00 (0.91, 1.10) *p*=0.897
7	3629 (8.9)	1735 (7.0)		1.44 (1.32, 1.57)	0.96 (0.85, 1.10) *p*=0.377
8	2911 (7.2)	1158 (4.7)		1.73 (1.57, 1.92)	1.01 (0.88, 1.20) *p*=0868
9	1106 (2.7)	452 (1.8)		1.69 (1.45, 1.96)	0.93 (0.75, 1.10) *p*=0.345
10	580 (1.4)	250 (1.0)		1.60 (1.31, 1.69)	0.78 (0.59, 1.10) *p*=0.021
**Self-rated mental health cognition and mood)**			<0.000		
Excellent	8057 (19.8)	6092 (24.6)		1	1
Very Good	13078 (32.2)	8950 (36.1)		1.10, (1.04, 1.17)	1.04 (0.96, 1.15), *p*=0.215
Good	11924 (29.3)	6286 (25.3)		1.43 (1.35, 1.52)	1.14 (1.04, 1.25), *p*>0.000
Fair	6009 (14.8)	2781 (11.2)		1.63 (1.52, 1.76)	1.02 (0.91, 1.15) *p*=0.609
Poor	1279 (3.1)	545 (2.2)		1.77 (1.55, 2.04)	1.11 (0.89, 1.36) *p*=0.213
No Answer	308 (3.1)	155 (0.6)		1.50 (1.16, 1.95)	0.97 (0.70, 1.33) *p*=0.778

Table 1 Legend: Distributions, central tendency, spread, unadjusted odds ratios and adjusted odds ratios of sample demographics, pre-infection symptom totals, and pre-infection functional status indicators.

* Run with Yeats’ continuity correction.

** From the “Finding of functional performance and activity” EHR observations.

**Table 2.** Pre-infection prevalences and odds of common post-COVID symptoms by long COVID group.

Long COVID symptom/condition group	*Recovered n(%)*	*Long COVID cases n(%)*	OR (99% CI) *P*	AOR (99% CI) *P*
Joint Pain	7626 (30)	25675 (62)	3.74 (3.58, 3.91) 0.00	1.32 (0.82, 2.11) 0.13
Chest Pain	5054 (20)	19400 (47)	3.20 (3.34, 3.68) 0.00	1.03 (0.64, 1.64) 0.89
Abdominal	5237 (21)	18803 (45)	3.17 (3.02, 3.33) 0.00	1.06 (0.66, 1.70) 0.75
Sleep	3902 (15)	18241 (44)	4.29 (4.07, 4.52) 0.00	1.53 (0.96, 2.45) 0.02
Anxiety	4148 (16)	17717 (43)	3.80 (3.61, 3.99) 0.00	1.36 (0.85, 2.18) 0.09
Depression	3634 (14)	16639 (40)	3.99 (3.79, 4.21) 0.00	1.31 (0.81, 2.10) 0.15
Cough	3977 (16)	15833 (38)	3.31 (3.15, 3.49) 0.00	1.08 (0.68, 1.74) 0.66
Dyspnea	3308 (13)	14670 (35)	3.64 (3.44, 3.45) 0.00	1.13 (0.70, 1.81) 0.51
Fatigue	3339 (13)	14172 (34)	3.42 (3.23, 3.61) 0.00	1.11 (0.70. 1.79) 0.54
Rash/Skin	2965 (12)	11488 (28)	2.86 (2.70, 3.04) 0.00	1.27 (0.79, 2.03) 0.19
Headache	2603 (10)	11051 (27)	3.16 (2.97, 3.36) 0.00	0.99 (0.61, 1.59) 0.95
Dizziness	2398 (9)	10388 (25)	3.19 (3.00, 3.40) 0.00	1.03 (0.64, 1.66) 0.86
Diarrhea	2076 (8)	9273 (22)	3.22 (3.01, 3.45) 0.00	1.07 (0.67, 1.71) 0.72
Muscle Pain	1253 (5)	6528 (16)	3.60 (3.31, 3.92) 0.00	1.16 (0.72, 1.86) 0.44
Palpitations	1469 (6)	6308 (15)	2.91 (2.69, 1.71) 0.00	1.14 (0.71, 1.84) 0.48
Fever	1390 (5)	5537 (13)	2.63 (2.45, 2.88) 0.00	0.89 (0.55. 1.43) 0.52
Cognition	1117 (4)	5131 (12)	3.03 (2.78, 3.32) 0.00	1.01 (0.63, 1.63) 0.96
Paraesthesia	1169 (5)	5111 (12)	2.88 (2.64, 3.14) 0.00	1.08 (0.67, 1.74) 0.67
Tachycardia	1225 (5)	4946 (12)	2.68 (2.46, 2.92) 0.00	0.85 (0.53, 1.37) 0.39
Menstrual Disorder	1475 (6)	4304 (10)	1.87 (1.73, 2.03) 0.00	1.07 (0.66, 1.73) 0.71
Musculoskeletal Chest Pain	648 (3)	3220 (8)	3.20 (2.87, 3.60) 0.00	1.09 (0.67, 1.77) 0.66
ME/CFS (Chronic Fatigue)	430 (2)	2228 (5)	3.33 (2.90, 3.83) 0.00	1.03 (0.63, 1.70) 0.87
Sexual Dysfunction	231 (1)	847 (3)	2.26 (1.86, 2.75) 0.00	0.99 (0.59, 1.66) 0.97

Table legend: Percent of participants with versus without long COVID with at least one pre-infection occurrence of common long COVID symptoms, with unadjusted and adjusted odds ratios of developing long COVID given each symptom/condition.

## Data Availability

This study used data from the *All of Us* Research Program’s Controlled Tier Dataset version 7, available to authorized users on the Researcher Workbench.
