# Supplementary material for: Long COVID risk by pre-infection symptoms and functional status: A retrospective cohort study of data from the All of Us Research Program
Source: medRxiv. 2025 Aug 12:2025.08.07.25333259. Preprint. [Version 1] doi: 10.1101/2025.08.07.25333259 (PMC12363706; doi:10.1101/2025.08.07.25333259)
Supplement: Supplement 1 [file NIHPP2025.08.07.25333259v1-supplement-1.pdf]

medRxiv preprint doi: https://doi.org/10.1101/2025.08.07.25333259; this version posted August 12, 2025. The copyright holder for this preprint (which was not certified by peer review) is the author/funder, who has granted medRxiv a license to display the preprint in perpetuity. This article is a US Government work. It is not subject to copyright under 17 USC 105 and is also made available for use under a CC0 license.

Supporting information

S1 File. Appendices A-E. Appendices with expanded description of cohort discovery; disease, symptom, and functional indicators; quantitative analysis of bias; model fitting notes; expanded results.

S1 Table A.1. Identification of cohort - COVID-19 illness indicators. All laboratory observations, COVID-19 Participant Experience (COPE) survey item responses, and diagnostic codes in SNOMED vocabulary indicating either SARS-CoV-2 infection or COVID-19 illness. Generated using queries for lab values, survey item responses, and diagnostic code incidences via the All of Us Researcher Workbench dataset builder.

S2 Table A.2. Long COVID symptoms/conditions used in Cohort discovery and classification. Standard concept names, concept codes, source vocabularies, and included sub-concepts of long COVID symptoms queried via the *All of Us* Researcher Workbench dataset builder. Hierarchical relationships are as organized in the Athena relational database of the Observational Health Data Sciences and Informatics (OHDSI) and queried from the Observational Medical Outcomes Partnership – Common Data Model (OMOP – CDM) table structures.

S3 Table D.1. Variables for model. Demographic, disease, pre-infection symptom, and pre-infection function variables (names, sources, and formats) used as covariates in regression models. Pre-infection variables are based on entry in the medical record between five years and four weeks prior to first infection date. Each variable's intercept value or level is noted.

S1 Fig C.1. Distribution of propensity scores for pre- versus post-infection enrollment. Jitter point horizontal arrays showing the overlapping ranges in propensity for being in the long

medRxiv preprint doi: https://doi.org/10.1101/2025.08.07.25333259; this version posted August 12, 2025. The copyright holder for this preprint (which was not certified by peer review) is the author/funder, who has granted medRxiv a license to display the preprint in perpetuity. This article is a US Government work. It is not subject to copyright under 17 USC 105 and is also made available for use under a CC0 license.

COVID group between the unmatched versus matched pre-infection enrollees ('control') and post-infection enrollees ('treated'; excluded for the present study's analysis). After matching pre- and post-enrollment participants, about a 45% overlap is seen between these two groups, indicating that participants may differ in one or more key demographic and disease aspects relating to when they enrolled in the study.

S2 Fig C.2. Standardized Mean Difference of demographic and disease characteristics by pre- versus post-infection enrollment.

S3 Fig. C.3. Distribution of propensity scores for 28-day versus 90-day symptom onset date. Overlaps in propensity score between the participants classified as cases at 28 days ('control') and those at 90 days ('treated') is visualized in Figure S.C.3. There was no overlap between cases and controls by the 90-day alternative classification scheme (distance = 1.0).

S4 Fig. C.4. Standardized Mean Difference of demographic and disease characteristics by 28-day versus 90-day symptom onset date

S5 Fig. E.1. Time of first infection, by long COVID group. Histogram of the number of participants with (darker blue) versus without (lighter blue) long COVID by first infection date through July 2022. The *Y* axis plots the number of participants ascending from zero (bottom) to over 1,500 (top); the *X* axis is a timeline from (left to right) January 1 2020 through July 31 2022. Both groups show a similar profile; they begin with a steep spike in infections about

medRxiv preprint doi: https://doi.org/10.1101/2025.08.07.25333259; this version posted August 12, 2025. The copyright holder for this preprint (which was not certified by peer review) is the author/funder, who has granted medRxiv a license to display the preprint in perpetuity. This article is a US Government work. It is not subject to copyright under 17 USC 105 and is also made available for use under a CC0 license.

March 2020, a large and prolonged peak between April and September 2020, and briefer peaks between about October 2020 – January 2021 and in January 2022.

S6 Fig. E.2. Proportion of sample with each pre-infection long COVID symptom, by group. Bar graph of the percentage of long COVID cases (light bars) and recovered participants (dark bars) with at least one pre-infection mention of each long COVID symptom/condition within the five years preceding their first COVID infection. The cases show between three to five times greater prevalence of every pre-infection symptom/condition, however none of these symptoms were significant predictors of long COVID when adjusted for demographics, disease factors, and pre-infection functional status.

S4 Table E.1. Correlation matrix of pre-infection symptoms. Table of Pearsons *R* correlation coefficients between pre-infection symptoms.

S7 Figure E.3. Symptom correlations grouped by first principal components analysis. Heat map of first principle components analysis of pre-infection symptom correlations reported in Table E.1. Darker green colors indicate stronger correlations, and white corresponds to no correlation.
